# Systematic Cross-biospecimen Evaluation of DNA Extraction Kits for Long- and Short-read Multi-metagenomic Sequencing Studies

**DOI:** 10.1016/j.gpb.2022.05.006

**Published:** 2022-06-06

**Authors:** Jacqueline Rehner, Georges Pierre Schmartz, Laura Groeger, Jan Dastbaz, Nicole Ludwig, Matthias Hannig, Stefan Rupf, Berthold Seitz, Elias Flockerzi, Tim Berger, Matthias Christian Reichert, Marcin Krawczyk, Eckart Meese, Christian Herr, Robert Bals, Sören L. Becker, Andreas Keller, Rolf Müller, The IMAGINE Consortium

**Affiliations:** 1 Institute of Medical Microbiology and Hygiene, Saarland University, D-66421 Homburg, Germany; 2 Clinical Bioinformatics, Saarland University, D-66123 Saarbrücken, Germany; 3 Department of Human Genetics, Saarland University, D-66421 Homburg, Germany; 4 Helmholtz Institute for Pharmaceutical Research Saarland, D-66123 Saarbrücken, Germany; 5 Clinic of Operative Dentistry, Periodontology and Preventive Dentistry, Saarland University, D-66421 Homburg, Germany; 6 Department of Ophthalmology, Saarland University Medical Center, D-66421 Homburg, Germany; 7 Department of Medicine II, Saarland University Medical Center, D-66421 Homburg, Germany; 8 Department of Internal Medicine V – Pulmonology, Allergology, Intensive Care Medicine, Saarland University, D-66421 Homburg, Germany

**Keywords:** Whole-genome analysis, Comparative genomics, Short-read sequencing, Long-read sequencing, DNA extraction, Metagenomics

## Abstract

High-quality **DNA extraction** is a crucial step in metagenomic studies. Bias by different isolation kits impairs the comparison across datasets. A trending topic is, however, the analysis of multiple metagenomes from the same patients to draw a holistic picture of microbiota associated with diseases. We thus collected bile, stool, saliva, plaque, sputum, and conjunctival swab samples and performed DNA extraction with three commercial kits. For each combination of the specimen type and DNA extraction kit, 20-gigabase (Gb) metagenomic data were generated using **short-read sequencing**. While profiles of the specimen types showed close proximity to each other, we observed notable differences in the alpha diversity and composition of the microbiota depending on the DNA extraction kits. No kit outperformed all selected kits on every specimen. We reached consistently good results using the Qiagen QiAamp DNA Microbiome Kit. Depending on the specimen, our data indicate that over 10 Gb of sequencing data are required to achieve sufficient resolution, but DNA-based identification is superior to identification by mass spectrometry. Finally, long-read nanopore sequencing confirmed the results (correlation coefficient > 0.98). Our results thus suggest using a strategy with only one kit for studies aiming for a direct comparison of multiple microbiotas from the same patients.

## Introduction

In the past decade, microbiome research has become a trending topic with an exponential increase of available data [1]. Researchers worldwide acknowledge the importance of the human microbiome for health [2] regarding a variety of diseases, with the gut microbiome taking a leading role [3]. Recently, the link between a healthy gut microbiome influenced by a Mediterranean diet and cardiometabolic disease risk has been found [4]. In addition, the gut microbiome of Parkinson’s disease patients has also been associated with intestinal inflammation [5]. Next to the gut microbiome, the microbiome of the respiratory tract has been studied extensively. For example, it has been previously shown that certain bacteria are associated with chronic rhinosinusitis. Bachert et al. [6], as well as Olzowy et al. [7], detected overgrowth of *Corynebacterium*, *Curobacteria*, *Pseudomonas*, *Staphylococcus*, and *Haemophilus influenzae* in patients with chronic rhinosinusitis compared to the healthy respiratory microbiota. There is accumulating evidence that microbiome research should also identify commensal bacteria and investigate their potential to protect from diseases. Several species are already known to synthesize compounds that inhibit the growth of pathogenic bacteria, thereby establishing a crucial balance within the microbiome. Besides the intended effects on pathogenic bacteria, antibiotic therapy also affects commensal bacteria, and may facilitate overgrowth of potentially dangerous microorganisms, as it is frequently seen in *Clostridioides difficile* infection, a common intestine complication after previous antibiotic treatment [8]. How is the growth of pathogens suppressed under normal conditions? During a co-infection, *Pseudomonas aeruginosa* produces rhamnolipids, which disperse the biofilms of sulfate-reducing bacteria and, additionally, are effective against the biofilms of opportunistic pathogens such as *Escherichia coli* and *Bacillus subtilis* [9]. *Staphylococcus lugdunensis* has been found to produce lugdunin, which is a recently discovered thiazolidine with antibiotic activity. Lugdunin inhibits the growth of the opportunistic pathogen *Staphylococcus aureus* [10]. Furthermore, certain lactic acid bacteria are known to produce a variety of secondary metabolites which inhibit the growth of other bacteria, such as bacteriocins, hydrogen peroxide, and diacetyl [11].

Bacteria have evolved for 4.3 billion years, and their metabolism and entire biosynthesis have perfectly adapted to their environments. They constantly fight for nutrients and space, trying to inhibit the growth of their competitors which renders them the perfect target for searching novel natural compounds to fight bacteria-associated diseases [12]. Also, in the sustainable development of new antibiotics, microbiota plays an essential role [13].

All these aspects can be discovered by examining the human microbiome of various compartments of the body by extracting the whole-genome DNA of clinical samples while depleting the human DNA. The usage of the extracted DNA for next-generation sequencing (NGS) can then shed light on all microorganisms that are in the native sample. This very precise method can be augmented with microbiological cultivation of the same samples. Which bacteria are cultivatable, and also during routine diagnostics which are only detectable by sequencing the native samples?

Many steps in the process of sample collection, DNA extraction, sequencing, and data analysis can introduce significant bias. One example is the stool collection kits used that already affect the reported microbial compositions [14]. Likewise, in oral microbiomes, bias is known and addressed [15]. The extraction of the whole-genome DNA is a crucial step. It is evident that the topic of comparing different DNA extraction kits is essential and thus has become an evolving field of research. For different specimen types, respective protocols have been compared, *e.g.*, for breast milk [16], stool [17], skin [18], vaginal swabs [19], sputum [20], postmortem eye tissue [21], nasal washes [22], and meconium [23]. As for one specific sample type, the most suitable DNA extraction method has been evaluated over several studies, but an analysis of different DNA extraction kits on their suitability for a variety of sample types has, to our knowledge, not been performed yet. It is interesting, however, to analyze various microbiomes without causing bias due to the use of different extraction protocols, to understand the complexity and connectivity of microbiomes at different body sites in health and disease. Analyses of different biospecimens yield inconsistent results, which renders the selection of the very best protocol challenging. While for studies on single specimen types the best kit for the respective specimen can be selected, multi-microbiome studies potentially suffer from bias if different kits are used.

**Figure 1:**
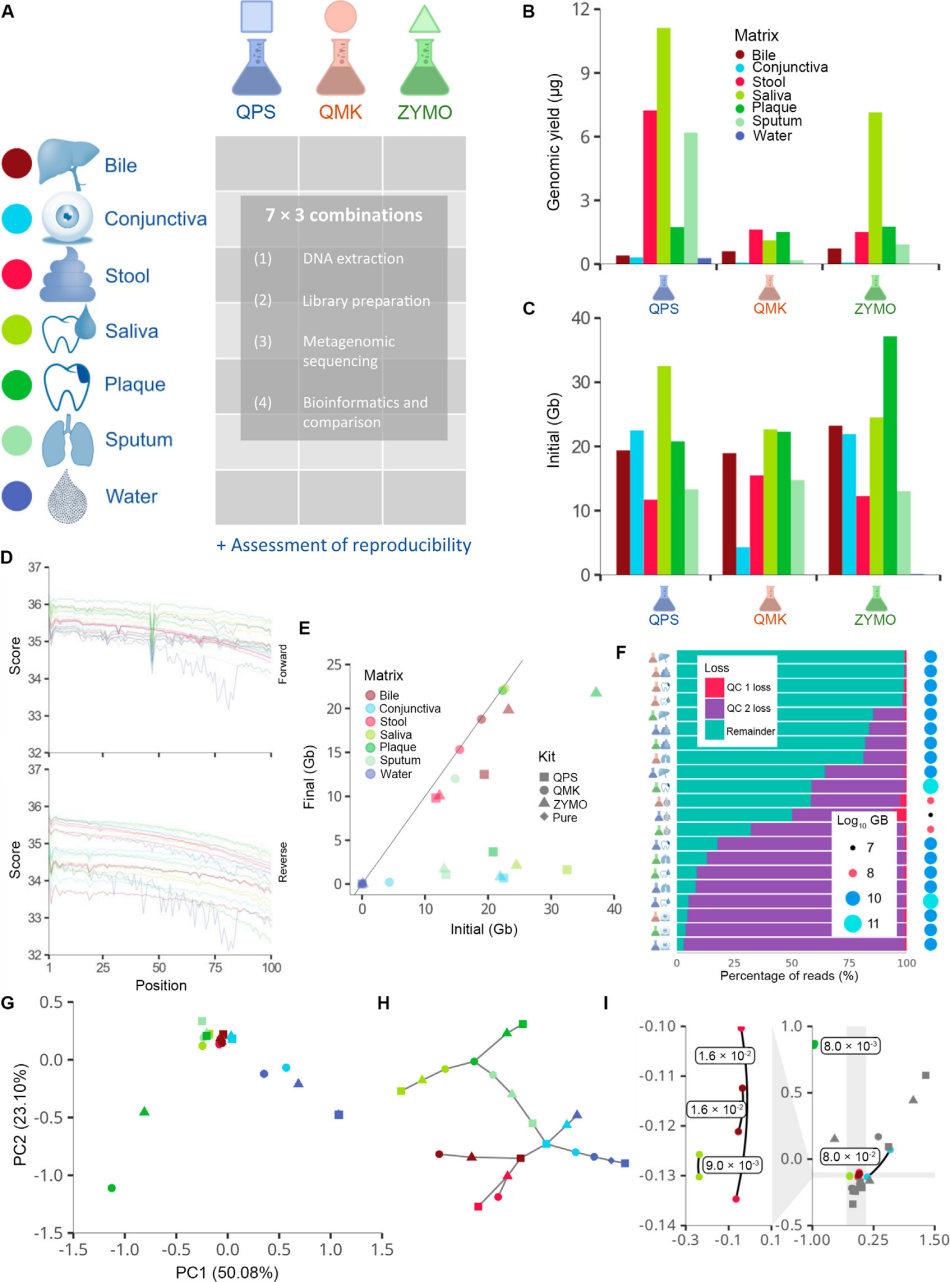
**Study setup and QC** **A.** For six specimen types and water, we performed DNA extraction using three different commercial kits. Following library preparation and sequencing, the metagenomes were evaluated and compared to each other. **B.** The DNA yield of the different specimen types with different kits is given as a bar diagram. **C.** Comparison of the raw sequencing output in Gb before QC. **D.** Q30 values of the raw sequencing reads. Colors indicate the various biospecimens, in line with (A). **E.** Scatter plot of the raw reads to the reads obtained after QC. Shapes represent the different kits, and colors represent the different biospecimens. **F.** Percentage of reads filtered in the different QC steps and remaining dataset size. QC 1 mostly captures loss attributed to read quality, while QC 2 focuses on contamination by host sequences. **G.** Principal component analysis of the different samples and kits using the Mash distances after QC. Shapes represent the different kits, and colors represent the different biospecimens. **H.** Minimum spanning tree of the Mash distances after QC. Shapes represent the different kits, and colors represent the different biospecimens. **I.** Recomputed embedding displaying Mash distances between replicates. Grey points are without replicates. Colors indicate biospecimens. QC, quality control; Gb, gigabase; PC, principal component; QPS, Qiagen DNeasy PowerSoil Pro; QMK, QiAamp DNA Microbiome Kit; ZYMO, ZymoBIOMICS DNA Miniprep Kit.

To understand microbiota in health and disease, multi-metagenomic studies that combine the microbiota from many samples of the same patients are however promising. We thus set out to identify a commercially available DNA extraction kit that is suitable to be used on such diverse biospecimens (Figure 1A). Here, we presented the data on the comparative extraction efficiency and sequencing quality obtained by whole-genome sequencing for six types of clinical samples (conjunctival swabs, stool, saliva, interdental plaque, bile, and sputum) after DNA extraction with three commercial kits, including 1) Qiagen DNeasy PowerSoil Pro (QPS; Qiagen, Hilden, Germany), 2) Qiagen QiAamp DNA Microbiome Kit (QMK; Qiagen, Hilden, Germany), and 3) ZymoBIOMICS DNA Miniprep Kit (ZYMO; Zymo Research Corp, Irvine, CA). QMK includes the advantage of host DNA depletion, presumably without causing taxonomic bias, which is a crucial step during DNA extraction for biospecimens such as skin and conjunctival swabs, for which more host material than bacterial mass is expected. This additional processing step might be a potential explanation for the increased price of QMK in comparison to the two competitor kits tested in this study. In contrast to the novel QMK, we tested ZYMO and QPS that have both been used frequently in regard to microbiome analysis [24], [25], [26], [27]. We followed a staged approach. We first performed a total of 108 DNA extractions and then chose the most promising samples for library preparation and sequencing. After evaluation of the sequencing data, we performed replicates for the best DNA extraction kit to analyze the reproducibility.

## Results

### DNA yield and sequencing quality vary between extraction kits and specimen types

As a first aspect, we compared the DNA yield and sequencing output for the different sample types and DNA extraction kits. The results showed that the DNA amount and concentration varied substantially between the different setups (Figure 1B, Figure S1F). It is known and expected that the different sample types — each with a different human background — lead to varying results in terms of reads and read quality. In line with the yield of DNA, the number of raw reads from the sequencing was likewise diverse (Figure 1C). Here again, the DNA extraction kits had a limited influence as compared to specimen types. However, the read quality in terms of Q30 value matched well, independent of the specimen type and DNA extraction kit, indicating that from all combinations interpretable microbiomes can be extracted (Figure 1D). The number of reads prior to and following quality control filtering generally correlated well for the different kits and sample types. Again, the fraction varied with the different specimen types depending on the expected human background, *e.g.*, introduced by human immune cells and human epithelial cells in saliva and conjunctiva, respectively (Figure 1E). This fact became more evident when considering the lost read fraction in the quality control steps. Again, independent of the kit, the conjunctival swab samples yielded only a fraction of 5% of all reads after quality control, dominated by the mapping of reads to the human genome (Figure 1F). Focusing on the fractions remaining after quality control, the QMK kit retrieved the highest amount of metagenomic information for each specimen type. However, the quantitative aspects were not the only criteria relevant for the selection of a kit, but also the composition of contents. Accordingly, we computed a 2-dimensional embedding using multidimensional scaling based on the Mash distances between samples (Figure 1G). Both, the embedding and the minimum spanning tree of the samples based on the mash distance, confirmed the general considerations: the kit has a limited influence on the output as compared to the difference introduced by the specimen types (Figure 1H). To provide further evidence for this behavior, we carried out technical replicates for five different QMK samples, demonstrating a high reproducibility of metagenomic measurements (Figure 1I).

In the light of the results in this section we might conclude that the variability introduced by the kits is so limited as compared to the difference between sample types and that for each specimen the very best kit might be selected even when multi-microbiome studies are performed. The high-level results, however, also call for a higher resolution analysis of the substantial metagenomic datasets.

### Metagenomes vary strongly between different DNA extraction kits, yet stronger between different specimen types

**Figure 2:**
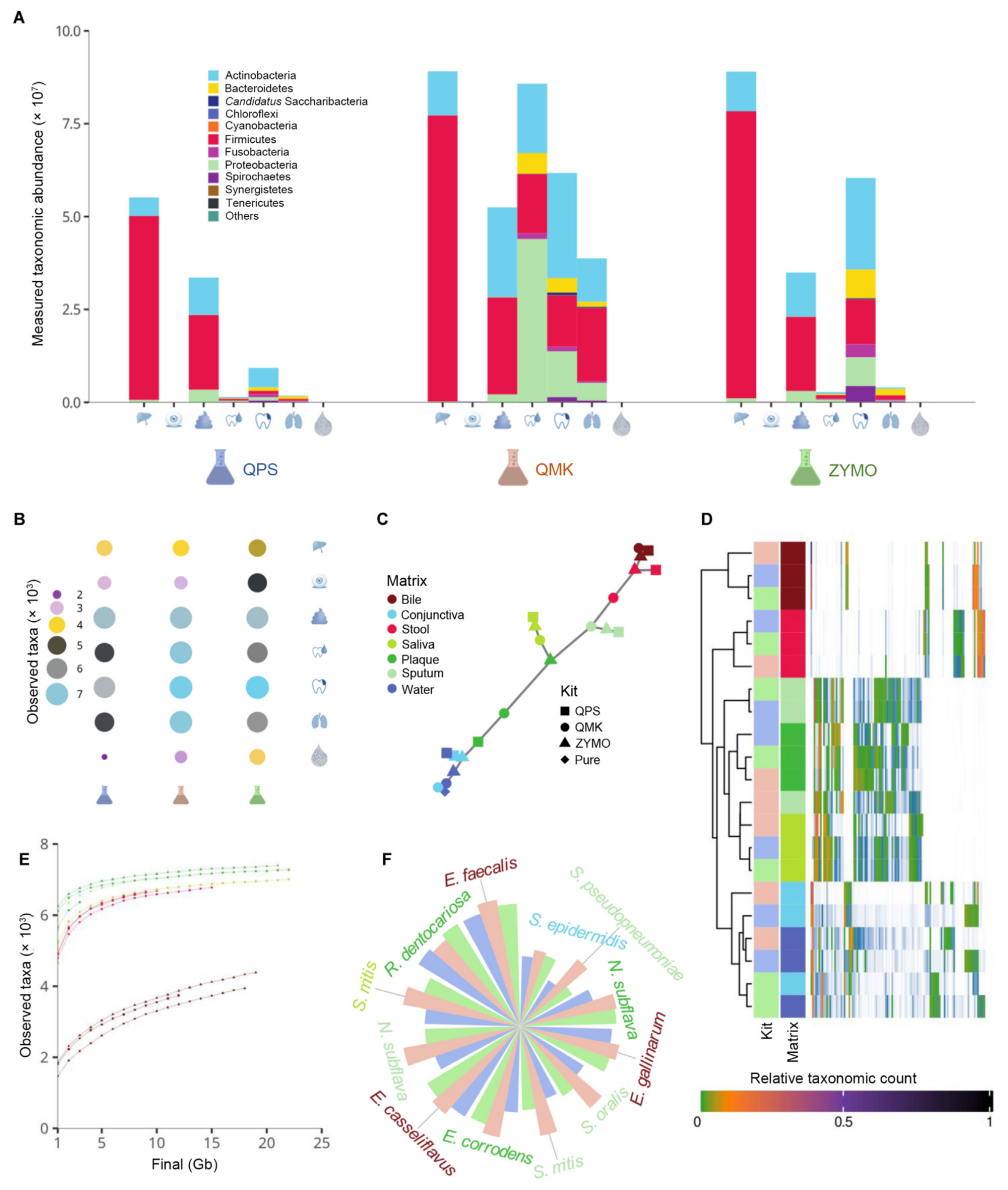
**Diversity of microbiota** **A.** Bar plot presenting the composition of bacterial microbiota with respect to different phyla. The color codes represent the phyla. Specimen types corresponding to each DNA extraction kit are in the following order: bile, conjunctival swab, stool, saliva, plaque, sputum, and water (left to right). **B.** For the seven biosamples, the observed unfiltered alpha diversity is presented. The color and bubble size correspond to the alpha diversity. Large and blue bubbles match samples with highest alpha diversity, and the small and purple bubbles match samples with lowest alpha diversity. **C.** Minimum spanning tree on the bacterial species level. Jaccard distance served as distance measure. Shapes represent the different kits, and colors represent the different biospecimens. **D.** Heatmap representing the abundance of different species clustered with respect to the sample type. Only species with a relative abundance above 1% were considered. Colors used to represent different kits are green for ZYMO, blue for QPS, and pink for QMK, in line with (A). Each biospecimen (matrix) is represented by a different color consistent with (C). Relative taxonomic counts are depicted in green for 0, black for 1, and shades of green, orange, and violet with increasing relative taxonomic counts between 0 and 1. **E.** Fraction of observed taxa with respect to the sequencing depth computed on sub-sampled decontaminated reads. Shapes represent the different kits, and colors represent the different biospecimens. **F.** Barplot displaying unnormalized counts in the NGS experiment for the different taxonomies that were detected by mass spectrometry. Font colors indicate specimen types, in line with (C). Bar colors represent the different kits, matching (A). NGS, next-generation sequencing.

First, we computed the bacterial phyla and families contained in the different samples to get an overview of the taxonomic profile (Figure 2A, Figure S1A). Overall, the large quantitative differences in data yield reflect abundance counts. Again, strong differences in relative composition were present for the specimen types. A more detailed consideration revealed a low relative amount of Proteobacteria for several ZYMO- and QPS-extracted samples compared to those extraced by QMK. Especially, the relative portion of Firmicutes decreased in the QMK-extracted samples as compared to those extraced by the other two kits. Mostly Proteobacteria measurements profited from this shift, which was most pronounced in saliva.

### The alpha diversity crucially depends on the DNA extraction kit and the sequencing depth

Quantitative differences do not necessarily translate to qualitative differences. Accordingly, we investigated the alpha diversity of the various samples. Again, specimen types dominated the overall signal. For bile, conjunctiva, plaque, and water specimens, the ZYMO kit measured the highest number of differing taxonomies (Figure 2B). In case data analysis accounts for signals found in negative controls, the alpha diversity measured in ZYMO began to fall in line with the other two kits (Figure S1B). Despite high fluctuations in alpha diversity and total abundance across kits, bacterial species information recaptured most of the structure previously identified from read information alone, confirming the quality of the taxonomic profiling analysis (Figure 2C, Figure S1C). Investigation of the beta diversity based on dimensionality reduction showed a tendency to group QMK-extracted saliva and sputum samples with plaque samples (Figure S1E). Hereby, the comparably low bacterial abundances of the other two methods may act as a confounding factor. Consistent with the previous minimum spanning trees, we observed a clustering of specimen types into three major categories: 1) the close to sterile water and conjunctiva, 2) the digestive system-focused bile and stool, and 3) the oral cavity specimens saliva and plaque where sputum integrates. Looking closer into the clustering, it is clearly visible that a minority of species contributes a majority of the signal (Figure 2D, Figure S1D). Nevertheless, often rare taxonomies of minimal statistical weight may also be of interest for the analysis due to the potential harboring of *e.g.*, virulence factors. Therefore, to analyze the feasibility of finding rare species in the various environments, we further investigated the number of identified taxonomies changing with sampling depth by doing *in-silico* downsampling (Figure 2E). Hereby, we noted that the kits seemed to converge at a similar rate to their asymptotic behavior. This point of convergence was reliably achieved at around 10 Gb after quality control. The maximal number of taxonomies seemed to differ mostly by specimen types, yet minor differences were also visible for kits, which is consistent with the previous finding. Mass spectrometry (MS)-based identification of 42 colonies indicated for 12 significant results that QMK generated highest counts for all but one confirmed species (Figure 2F). We noted that two species were not detected in our genomic data analysis at all, but were found during MS, which were *Veillonella rogosae* and *Capnocytophaga granulosa* in saliva and sputum, respectively.

### The composition of microbiota considerably varies between DNA extraction kits

**Figure 3:**
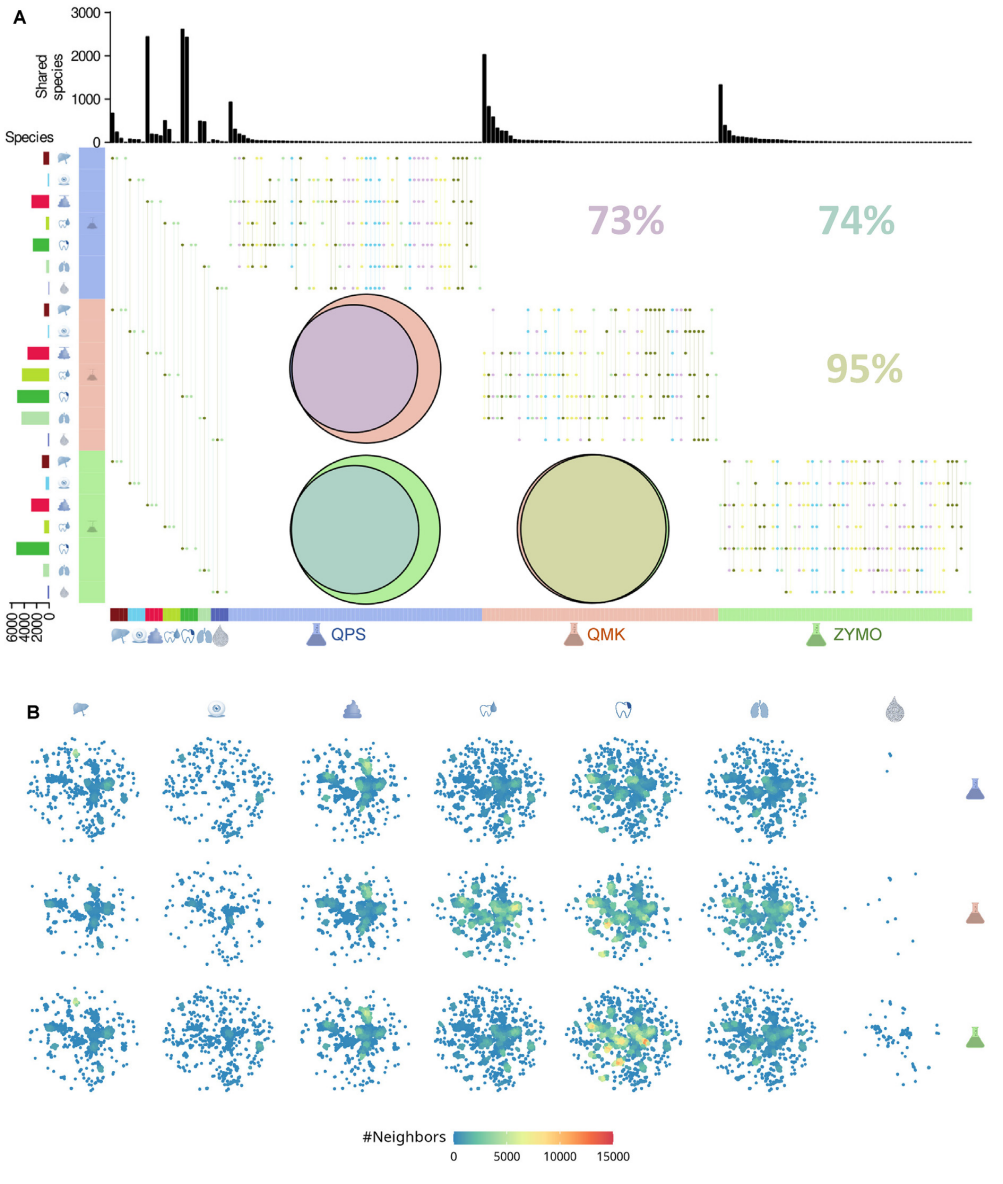
**Similarity of microbiota** **A.** Combination of ten upset plots each discussing identified species overlap by kit or specimen type. Bottom annotation indicates the aspect the upset plot focuses on, *i.e.*, which kit or specimen type is kept constant. Area proportional Euler diagrams below the diagonal capture the proportion of species identified independently of specimen type. Percentages above the diagonal indicate the overlap numerically. A species is considered identified after surpassing a low count threshold of 20 occurrences. **B.** Embedded microbiota. Each spot represents one scaffold with a length above 3 kb after an embedding of the *k*-mer spectra using UMAP. Colors are indicative of the point density in the respective area. UMAP, Uniform Manifold Approximation and Projection.

The taxonomic composition of a microbiome is often the key aspect to reveal during a metagenomic experiment. Hereby, the selected DNA extraction kit may play a crucial role on the qualitative findings of an experiment. While the previously discussed alpha diversity described the general number of different taxonomies captured by an experiment, it failed to discuss the exact nature of these differences. Therefore, we looked at the overlapping sets of detected species across both specimen types and kits (Figure 3A). Hereby, we selected a raw abundance count threshold to decide about the presence of a species instead of selecting by relative abundance, to also consider rare species in the analysis whose relative counts may undercut relative thresholds. We first discussed the common species of the individual specimen types. The largest intersecting set is usually the set encompassing all three kits. Only for sputum and water, the consensus was the largest for ZYMO and QMK. For the majority of time, ZYMO built the largest intersections, likely due to frequently constituting the largest stand-alone set. Next, we glanced at potential species that were found independently of input samples for the different kits. Here, the largest intersections were the ones with the largest initial sets. Due to higher measured bacterial abundances, QMK proposed four larger sets including sputum and saliva, whereas ZYMO and QPS only proposed stool and plaque as larger sets. Ignoring the underlying specimen types and aggregating the analysis, ZYMO and QMK had the largest number of species they detected in any specimen type.

Since taxonomic profiling is often limited by the quality and amount of reference organisms available, we further investigated ways to discuss potential differences in taxonomic composition between experiments that remain uncaptured by reference-based analysis. Hereby, we fell back on the core algorithm of BusyBee [28]. Accordingly, for reference-free analysis a Uniform Manifold Approximation and Projection (UMAP) embedding of normalized *k*-mer counts was computed on assembled scaffolds (Figure 3B). Visually, the embedding confirmed several findings of the previous taxonomic profiling. The overall density of the embeddings falls in line with the findings of the alpha diversities. Overall, it appears that ZYMO generates the highest density regions and is spreading all over the two-dimensional plane. While the embedding computed on QPS samples also scatters, there are fewer high-density regions. Last, QMK produces well defined regions of higher density. Moreover, the two clusters found in ZYMO water can be seen in all other samples except for the QPS water and QMK water. However, the left cluster also seems to disappear in QPS conjunctiva.

### Assembly quality depends on the specimen types and the DNA extraction kits

**Figure 4:**
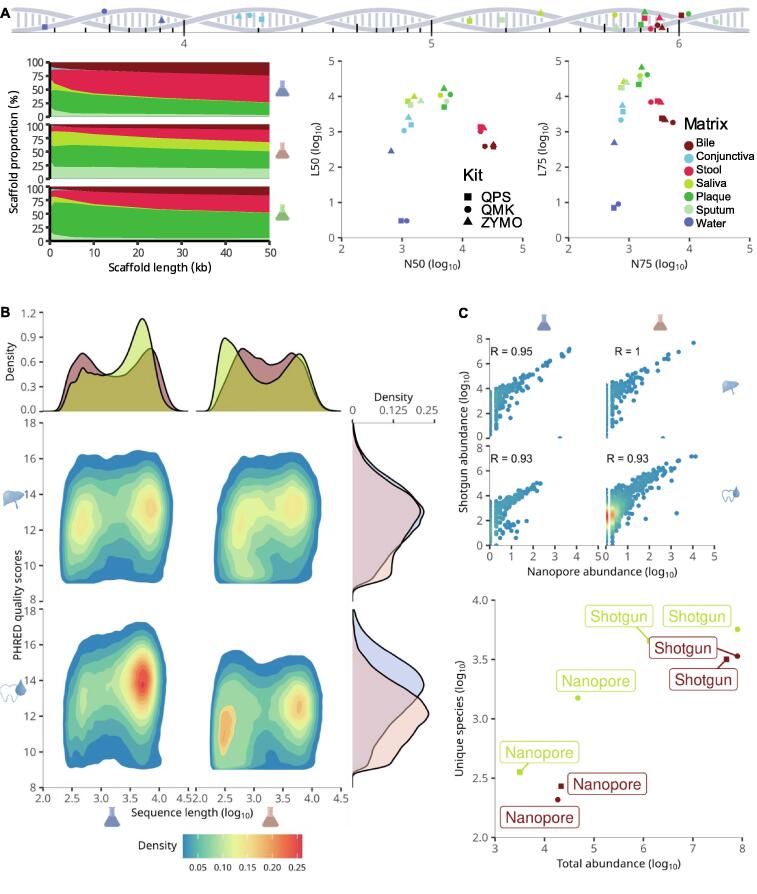
**Assemblyand nanopore comparison** **A.** Assembly quality. One-dimensional line showing the length of the longest scaffold for each assembly. Relative scaffold length distribution by kits together with their N50, N75, L50, and L75 values. **B.** Nanopore QC. Average PHRED scores indicate basecalling quality per read. Sequence length indicates length after basecalling of each read. Density plots on the right and top discuss the conditioned distributions for the different kits and specimen types. Visualized data are representative for data after filtering. **C.** Correlation plot indicating coherence between nanopore and shotgun sequencing taxonomic counts on bacterial species level without thresholding. Numerical values represent the rounded Pearson correlation before log scaling. The total number of measurements and unique different species for the different experiments are shown at the bottom.

For the previous reference-free analysis, assembly quality was comparably of minor importance due to the decomposition of assembled sequences into short *k*-mers. Yet, depending on further downstream analysis, the quality of metagenomic assembly may play a crucial role. Accordingly, we compared several assembly quality measures across kits and specimen types (Figure 4A). Considering length distribution, specimen types were mostly clustered together. However, for the three specimen types of saliva, plaque, and sputum, minor differences were visible with respect to kits, favoring QMK in N50 and N75 measures. Considering the proportion of scaffolds at changing length, QMK was the only kit where no specimen started to dominate after a given length. Last, ZYMO generated the longest assemblies in water and dominated L50 and L75 for water and conjunctiva.

### Taxonomic profiles are consistent across sequencing technologies

With the rising popularity of nanopore sequencing technology and the immense advantages it brings to metagenomics, in terms of assembly quality increase and interpretability, hybrid protocols combining shotgun and nanopore sequencing are continuously gaining in relevance. Correspondingly, the demands to kits supporting both protocols are favored. Since we previously demonstrated clustering behavior into three major clusters, we selected saliva and bile as representatives of the non-sterile specimen types and sequenced the same samples again with nanopore sequencing. Similarly, we removed the ZYMO kit for the experiment, due to our previous findings of a high number of false positives shown across several of our herein presented analyses. Quality control of nanopore reads of all four samples after filtering suggested minor differences between specimen types for both kits (Figure 4B). Pearson correlation between length and PHRED scores was around a low 0.1. Considering both, read length and average read quality scores, Wilcoxon rank sum tests across all reads indicated statistically significant differences between kits, conditioned on the specimen types for each sample (*P* < 1 × 10^−12^). After quality control, taxonomic profiling was performed to gauge the effects of interactions among kits, specimen types, and sequencing technologies (Figure 4C). As expected, the number of different identified bacterial species and overall abundance were a lot higher for shotgun sequencing due to the generation of false positives during profiling with short reads and the increased sequencing depth. Overall, the ordering of experiments based on uniquely quantified bacterial species remains unchanged across sequencing technologies. The only difference was that in bile, where QMK detected fewer unique species than QPS for the nanopore technology which may be linked to the difference in sampling depth and correlated total abundance. Glancing at the taxonomic profile on a bacterial species level, we observed strong correlations between nanopore and shotgun sequencing, for all tested kit and specimen combinations.

## Discussion

We evaluated three DNA extraction kits across six different specimen types and water to gauge their suitability for metagenomic experiments. We note that the QMK kit usually yields the highest amount of metagenomic information after host DNA removal. The depletion of human DNA is a significant advantage of QMK compared to ZYMO and QPS. This is consistent with the idea, to lyse all eukaryotic cells in a first step, followed by the degradation of eukaryotic DNA. Therefore, human DNA in particular is depleted during the first part of the DNA extraction with the QMK kit. During the second part, bacterial cells are lysed and the extracted DNA is purified.

Focusing on both, read information and metagenomic data analysis, we showed that the selection of the specimen type dominated the selection of the kit in signal strength. While for the difference between *e.g.*, water and stool, this result was to be expected, the same did not hold true for plaque and saliva samples. Further, we demonstrated the sensitivity of all kits by confirming a selection of taxa using MS. Considering specificity, we demonstrated, using a reference-based and reference-free method, that ZYMO appeared to contain most contamination, going hand in hand with the fact of ZYMO generating the samples with the highest relative amount of human contamination. This effect could be due to unsterile lysis tubes or columns for DNA extraction. However, we noted that no sample remained uncontaminated. The lowest contamination was shown for QPS. Especially in comparison to the QMK water sample, a lower contamination of the QPS sample can be explained by a general lower variety in identified bacteria species. Partly, bacteria in the environment, that contaminate the water samples might be harder to lyse, which the QPS kit might not have to offer. In contrast to the ZYMO and QPS kits, a pre-contamination of columns provided by the QMK kit is highly unlikely, as the special Qiagen ultra-clean columns were stored at 4 °C until being used for DNA extraction. Shifting focus away from taxonomic profiling onto assemblies, except for the ZYMO water sample, assemblies were of similar quality at first sight. Here we acknowledge that the scaffold length distribution is not the be-all and end-all of metagenomic assembly quality assessment; however, it is one of the more widely spread [29]. Last, for QPS and QMK we observed that overall, the results after metagenomic analysis are consistent across shotgun and nanopore sequencing. We note that our study is limited by the small sample size and the focus on bacterial microorganisms. Random sampling error may distort our findings. Thus, larger studies, including more replicates, are needed to confirm our results, and similar comparative studies should ideally also assess results for other pathogen classes, such as viruses or parasites [30].

To conclude, we recommend the QMK kit for samples with high eukaryotic host contamination, as it clearly has the least information loss upon host sequence removal. Moreover, if no detection threshold is set, QMK identifies generally more species than QPS, while not showing a strong contamination of sequencing results in sterile water as compared to ZYMO. In case host contamination is not an issue to consider, QPS may be recommended, since it shows the least overall contamination in sterile water.

## Materials and methods

### Sample collection

In brief, stool samples were collected by each participant using a paper toilet-hat and a sterile collection tube with an integrated spoon. Approximately 500 mg to 1 g of stool were collected. Plaque samples were collected using 12 disposable micro applicators (Catalog No. MSF400, Microbrush International, Grafton, WI). Three interdental spaces per quadrant were brushed, and all micro applicators were placed into a single ESwab transport tube (Copan Diagnostics, Brescia, Italy), including the ESwab Amies Medium (Copan Diagnostics). Saliva samples were collected using 50-ml sterile, conic falcon tubes. Participants were asked to release uninduced saliva into the sterile falcon tube for 5 min. Conjunctiva samples were obtained using a ESwab. The lower eyelid was everted, and the conjunctiva was swabbed throughout the entire length of the lower fornix three times. Afterwards, the swab was placed in the respective transport medium and the tube was frozen at −80 °C. Sputum was induced by 7 min of inhalation with 0.9% NaCl solution. After inhalation, the participant was asked to release sputum by coughing into a sterile collection tube. Bile samples were collected during a duodenoscopy by drawing 5 ml to 10 ml into a sterile syringe.

### DNA extraction

DNA was extracted from all samples using three different, commercially available DNA extraction kits: QPS, QMK, and ZYMO. For each kit, the DNA was extracted according to the manufacturer’s protocol. Briefly, 1 ml of sterile Milli-Q water was used for the negative control. The manufacturer’s protocol was followed, respectively. Fecal samples were weighed, and 250 mg of stool were used for DNA extraction with QPS and QMK, and 50 mg of stool were used for ZYMO, according to the manufacturer’s recommendation. For QPS and ZYMO, 1.5 ml of saliva samples were centrifuged for 5 min at 6000 *g* and the pellet was resuspended in the respective lysis buffer. For QMK, 1 ml of saliva was used directly. Interdental microbrushes and conjunctival swabs were vortexed rigorously in the eSwab Amies Medium for 3 min. The Amies Medium was then transferred to a 1.5-ml sterile Eppendorf tube and centrifuged for 5 min at 6000 *g* for further DNA extraction with QPS and ZYMO. The pellet was resuspended in the respective lysis buffer. For DNA extraction with QMK, the liquid Amies Medium was used directly. For DNA extraction with QPS and ZYMO, bile samples were vortexed rigorously and 2 ml of bile were transferred to a 2-ml sterile Eppendorf tube and centrifuged for 5 min at 6000 *g*. The supernatant was discarded, and the pellet was resuspended in the respective lysis buffer. To extract DNA from bile via QMK, 1 ml of bile was used directly. Sputum was mixed with Remel Sputasol (Oxoid L TD, Hants, England) in a 1:1 ratio. For QPS and ZYMO, 1.5 ml of sputum or sputasol was centrifuged for 5 min at 6000 *g* and the pellet was resuspended in the respective lysis buffer. For QMK, 1 ml of sample was used for DNA extraction without previous centrifuging. The mechanical lysis of bacterial cells was performed using the MP Biomedicals FastPrep-24 5G Instrument (FisherScientific GmbH, Schwerte, Germany). For ZYMO, the velocity and duration were adjusted to 6 m/s for 45 s three times with 30 s of storage on ice in between each lysis step. For elution of DNA during the last step of each DNA extraction kit, the following elution volumes were used: 1) QPS: 40 µl; 2) ZYMO: 20 µl; 3) QMK: 50 µl. The DNA concentration was determined via NanoDrop 2000/2000c (ThermoFisher Scientific, Wilmington, DE) full-spectrum microvolume UV–Vis measurements. For each sample type and each DNA extraction method tested, we used a total of one biological replicate for sequencing. However, DNA was isolated from a total of n = 10 biological replicates for saliva, interdental plaque, and stool, a total of n = 4 for bile, a total of n = 8 for sputum samples, and a total of n = 4 for conjunctival swabs. From all samples that we extracted DNA from, we selected the most promising samples for library preparation and sequencing. We chose those samples with the highest amount of DNA, least impurities, and least fragmentations. For all samples prepared with QMK we performed an n = 2 technical replicates for library preparation and sequencing.

### Library preparation

DNA libraries were prepared using the MGIEasy Universal DNA Library Prep Set (MGI Technologies, Shenzhen, China) according to the manufacturer’s recommendations. In general, 200 ng DNA was sheared into fragments using the M220 Focused-ultrasonicator (Covaris, Woburn, MA), followed by size selection using Agencourt AMPure XP beads (Beckman Coulter, Brea, CA). For low-biomass samples, such as the conjunctival swab and the sterile water control, the entire amount of isolated DNA was used as an input for the fragmentation procedure. The fragmented DNA was used for end-repairing and A-tailing. Next, adaptors containing specific barcodes were ligated to the 3′ and 5′ ends, and the ligation products were amplified by PCR. The concentration of the PCR products was measured using Qubit 1× dsDNA HS Assay Kit (ThermoFisher Scientific, Waltham, MA). In the following, 8 different barcoded samples were pooled in equal amount and circularized to generate the single-stranded DNA library. The concentration of the library was measured using Qubit ssDNA Assay Kit (ThermoFisher Scientific, Waltham, MA). Additionally to the different biospecimen samples, a sterile DNase/RNase-free water sample was prepared using the same procedure as for all samples.

### NGS

For the short-read sequencing, all libraries were sent to BGI Group for DNA nanoball (DNB) generation and paired-end sequencing (PE100) on the DNBSEQ-G400 instrument according to manufacturer’s instructions and recommendations.

### MinION library preparation and sequencing

Upon opening of the flow cell and again immediately prior to sequencing, flow cell pore count was measured using MinKNOW. Library preparation kits, flow cell, and other consumables used for the experiment are described in Table S1. DNA was quantified via Nanodrop 2000/2000c (ThermoFisher Scientific, Wilmington, DE) and the volume was determinated by using a pipette (Table S2). The library preparation was conducted according to the protocol “Native barcoding genomic DNA (with EXP-NBD 104)” provided by Oxford Nanopore Technologies (ONT), with the exception of the barcode ligation step and further the adapter ligation step for which the ligation mix was incubated for 15 min at room temperature instead of 10 min. The amount of initial DNA used for the barcoding kit was above 100 ng for the four specimen types corresponding to the DNA extraction kits shown in this study. In sum, the library consisted of 12 barcoded DNA samples. The barcoded DNA was stored at 4 °C for 3 days until adapter ligation. For barcoded libraries, volume-equal quantities of each sample were used for the final library. The amount of pooled barcoded DNA exceeded the recommended amount of 700 ng DNA by an additional 400 ng to reach a final DNA amount of about 1100 ng for adapter ligation (Table S3). For the last Agencourt Ampure XP bead clean-up step, short fragment buffer (SFB) was used. The completed library was loaded onto a R9.4 flow cell as per instructions given by ONT. Given the rapid advancement of protocols, chemicals, and the technology itself, data were generated with the most up-to-date methods and protocols available from ONT at the time of library preparation and sequencing. The Mk1B MinION device was used for data acquisition.

### Nanopore sequencing

MinION analysis was carried out at the Helmholtz Institute for Pharmaceutical Research Saarland (HIPS) at the Department Microbial Natural Products, Saarbrücken, Germany. The barcoded library, consisting of the metagenomic DNA samples, was generated in a S1 laboratory, whereas the sequencing of the samples was performed in the office. Sequencing methods performed simultaneous 1D sequencing of samples using native barcoding. The sequencing run was carried out over a time range of three days. At the time of use, the R9.4 spotON Flow Cell had a pore count exceeding the guaranteed level (> 800 pores) by the manufacturer. Pore count was measured by the MinKNOW software with a result of 808 pores. The majority (> 50%) of sequencing data were generated in the first 9 h of sequencing, corresponding to the time in which the first group of pores is actively sequencing. More than 99% of sequencing data were generated after 28 h of sequencing. The sequencing yield in a total number of estimated bases is displayed in Figure S2.

### Culturing of bacteria

All native samples were streaked out on four different agar plates: TSA with 5% sheep blood (TSA), MacConkey (MC), Columbia (Co), and Chocolate Blood (CB) agar plates (ThermoFisher Scientific, Wilmington, DE). All TSA, MC, and CB agar plates were incubated at 35.6 °C and 5% CO_2_ for a minimum of 18 h and a maximum of 24 h. Co agar plates were used for the cultivation of anaerobic bacteria and therefore incubated in an anaerobic environment for a minimum of 48 h.

### MS-based identification

Bacterial colonies, obtained by culturing on different agar plates, were spotted onto the MALDI-TOF target plate, followed by overlaying with 1 µl of α-cyano-4-hydroxycinnamic acid (CHCA) matrix solution (Bruker Daltonics), composed of saturated CHCA dissolved in 50% (v/v) of acetonitrile, 2.5% (v/v) of trifluoroacetic acid, and 47.5% (v/v) of LC-MS grade water. After drying at room temperature, the plate was placed into the Microflex LT Mass Spectrometer (Bruker Daltonics) for MALDI-TOF MS. Measurements were performed using the AutoXecute algorithm in the FlexControl software (v3.4; Bruker Daltonics). For each spot, 240 laser shots in six random positions were carried out automatically to generate protein mass profiles in linear positive ion mode with a laser frequency of 60 Hz, a high voltage of 20 kV, and a pulsed ion extraction of 180 ns. Mass charge ratio range (*m/z*) was measured between 2 kDa and 20 kDa. Bacterial species were identified by using the MALDI BioTyper software. Identification scores above 2.0 were considered a precise identification, scores between 1.7 and 1.99 were considered as possible species identification, and all identification scores below 1.7 were considered unsuccessful identification.

### Data analysis

First, quality control was performed with MultiQC (v1.9) [31] and fastp (v0.20.1) [32]. Next, NGS data were decontaminated of host sequences using kneaddata (v0.7.4). Decontaminated data were uploaded to the Sequence Read Archive (SRA) [33]. We counted the exact number of basepairs contained in the fasta files before the individual steps to get a detailed overview on the overall information content. Once the data were fully cleaned, Mash distances were computed on all remaining read information with Mash (v2.3) [34]. Taxonomic profiling was done with Kraken (v2.1.2) [35]. Optional downsampling of reads was performed with seqtk (v1.3). The PlusPF database release from 9/19/2020 was used as Kraken2 index. As an alpha diversity measure, we used either the observed number of different taxa or the Shannon index. As the beta diversity measure, the Jaccard index was computed. For clustering analysis, species with relative species abundance below 1% in all samples were removed. Samples were then clustered using Ward’s hierarchical agglomerative clustering in combination with the Euclidian distance measure. UMAP embeddings were computed on all scaffolds having a length over 3 kb. To this end, 5-mers of each scaffold were counted and assembled into a vector. Each vector was divided by its sum, scaled, and centered. The normalized counts were then passed to embedded using UMAP. Assemblies were computed with SPAdes (v3.15.2) using the --meta flag [36]. Scaffold quality assessment was made with MetaQUAST (v5.0.2) [37], enabling the splitting of scaffolds. Downstream analysis heavily relied on phyloseq (v1.36.0). Nanopore reads were basecalled with guppy (v5.0.7) [38] before undergoing taxonomic profiling.

## Ethical statement

All samples were collected at the Saarland University Medical Center, Germany, after having obtained written informed consent from all participants. The study was approved by the local ethics committee (Ärztekammer des Saarlandes) under reference 131/20.

## Data availability

Respecting the German Bundesdatenschutzgesetz, we uploaded the data after human read removal to the SRA of National Center for Biotechnology Information (NCBI). Preprocessed data can be found in SRA of NCBI (SRA: PRJNA802336), and are publicly accessible at https://www.ncbi.nlm.nih.gov/sra.

## CRediT author statement

**Jacqueline Rehner:** Methodology, Validation, Investigation, Writing - original draft. **Georges Pierre Schmartz:** Methodology, Software, Validation, Formal analysis, Data curation, Writing - original draft, Visualization. **Laura Groeger:** Methodology, Investigation, Writing - review & editing. **Jan Dastbaz:** Methodology, Investigation, Writing - review & editing. **Nicole Ludwig:** Supervision, Writing - review & editing. **Matthias Hannig:** Resources, Supervision, Writing - review & editing. **Stefan Rupf:** Resources, Supervision, Investigation, Writing - review & editing. **Berthold Seitz:** Resources, Supervision, Writing - review & editing. **Elias Flockerzi:** Supervision, Investigation, Writing - review & editing. **Tim Berger:** Investigation, Writing - review & editing. **Matthias Christian Reichert:** Supervision, Investigation, Writing - review & editing. **Marcin Krawczyk:** Resources, Supervision, Writing - review & editing. **Eckart Meese:** Resources, Supervision, Writing - review & editing. **Christian Herr:** Supervision, Investigation, Writing - review & editing. **Robert Bals:** Conceptualization, Methodology, Resources, Supervision, Project administration, Funding acquisition, Writing - review & editing. **Sören L. Becker:** Conceptualization, Methodology, Resources, Supervision, Project administration, Funding acquisition, Writing - review & editing. **Andreas Keller:** Conceptualization, Methodology, Software, Resources, Supervision, Project administration, Funding acquisition, Writing - review & editing. **Rolf Müller:** Conceptualization, Methodology, Resources, Supervision, Project administration, Funding acquisition, Writing - review & editing. All authors have read and approved the final manuscript.

## Competing interests

Georges Pierre Schmartz, Matthias Hannig, Stefan Rupf, Andreas Keller, and Rolf Müller are shareholders of MOOH GmbH.

## Supplementary material

Supplementary Figure S1 **Additional assessment of microbiota diversity A.** Identical plot to Figure 2A focusing on families instead of phyla. **B.** Identical visualization to Figure 2B with two changes in data analysis. First, the Shannon diversity is used as alpha diversity measure. Second, a decontamination procedure was used to remove contaminations found in the negative control samples. For each kit, all species with relative abundance over 0.01 were identified in the water samples. Those taxonomies were then blacklisted to not be included in the computation. **C.** Figure 2C implementing the decontamination procedure described in Figure S1B. **D.** Heatmap with clustering of Figure 2D integrating the decontamination procedure used in Figure S1B. **E.** Visualized beta diversity computed using PCoA based on the Bray-Curtis distance measure. **F.** Barplot shows the proportion of isolated DNA in ng/mg stool, ng/ml saliva or water, and ng/ml swab-medium mixture for the remaining samples. PCoA, principal coordinates analysis.

Supplementary Figure S2 **Sequencing yield in estimated bases across experiment time** The graph was plotted by Excel (Microsoft) with the throughput Excel file generated by MinKNOW.
