# Supplementary figures and images for: Systematic Cross-biospecimen Evaluation of DNA Extraction Kits for Long- and Short-read Multi-metagenomic Sequencing Studies
Source: Genomics Proteomics Bioinformatics. 2022 Jun 6;20(2):405–17. doi: 10.1016/j.gpb.2022.05.006 (PMC9684153; doi:10.1016/j.gpb.2022.05.006)

## Slide 1
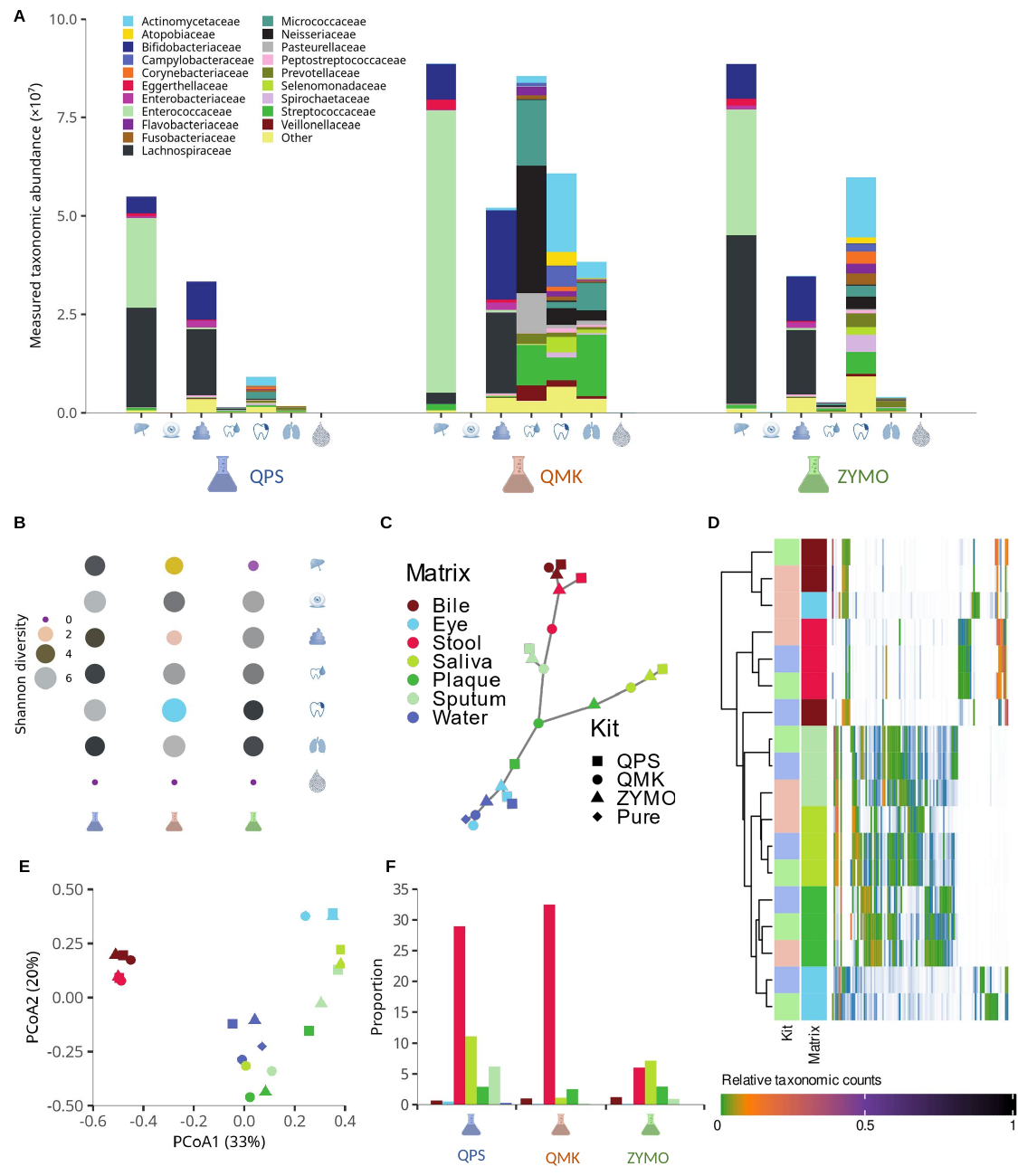

A
QMK
ZYMO
QPS
B
C
D
E
F
QPS
QMK
ZYMO

Supplement: Supplementary Figure S1 — Additional assessment of microbiota diversity A. Identical plot to Figure 2A focusing on families instead of phyla. B. Identical visualization to Figure 2B with two changes in data analysis. First, the Shannon diversity is used as alpha diversity measure. Second, a decontamination procedure was used to remove contaminations found in the negative control samples. For each kit, all species with relative abundance over 0.01 were identified in the water samples. Those taxonomies were then blacklisted to not be included in the computation. C. Figure 2C implementing the decontamination procedure described in Figure S1B. D. Heatmap with clustering of Figure 2D integrating the decontamination procedure used in Figure S1B. E. Visualized beta diversity computed using PCoA based on the Bray-Curtis distance measure. F. Barplot shows the proportion of isolated DNA in ng/mg stool, ng/ml saliva or water, and ng/ml swab-medium mixture for the remaining samples. PCoA, principal coordinates analysis. [file mmc1.pptx]

## Slide 1
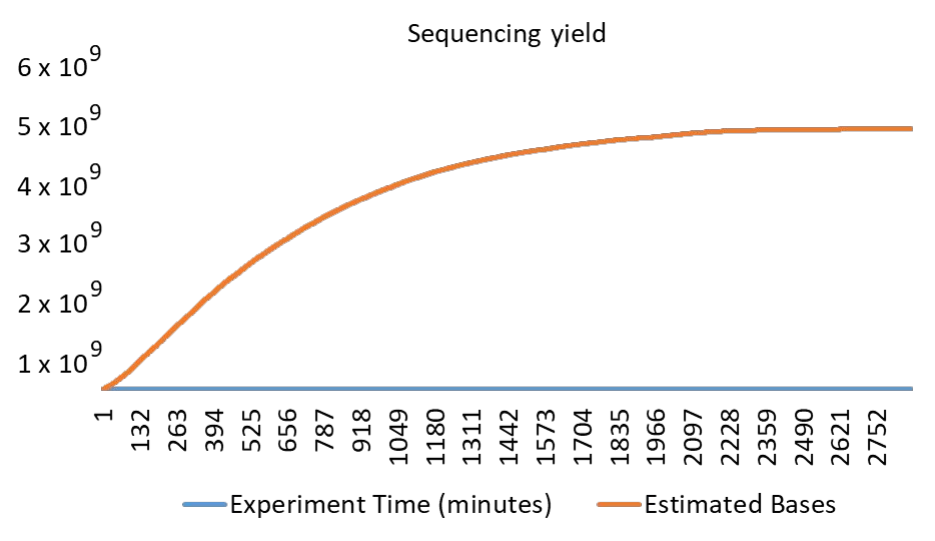

Supplement: Supplementary Figure S2 — Sequencing yield in estimated bases across experiment time The graph was plotted by Excel (Microsoft) with the throughput Excel file generated by MinKNOW. [file mmc2.pptx]
