# Supplementary material for: Systematic Cross-biospecimen Evaluation of DNA Extraction Kits for Long- and Short-read Multi-metagenomic Sequencing Studies
Source: Genomics Proteomics Bioinformatics. 2022 Jun 6;20(2):405–17. doi: 10.1016/j.gpb.2022.05.006 (PMC9684153; doi:10.1016/j.gpb.2022.05.006)
Supplement: Supplementary Table S1 [file mmc3.docx]

| **Materials & consumables** | **Company** |
| --- | --- |
| MinION Mk1B | Oxford Nanopore Technologies |
| Flow Cell (R9.4.1) FLO-MIN106D | Oxford Nanopore Technologies |
| Ligation Sequencing Kit 1D (SQK-LSK109) | Oxford Nanopore Technologies |
| Native Barcoding Expansions 1-12 (EXP-NBD104) | Oxford Nanopore Technologies |
| Flow Cell Priming Kit (EXP-NBD114) | Oxford Nanopore Technologies |
| Short Fragment Buffer (SFB) | Oxford Nanopore Technologies |
| Elution Buffer (EB) | Oxford Nanopore Technologies |
| Adapter Mix II (AMII) | Oxford Nanopore Technologies |
| Agencourt AMPure XP beads | Beckman Coulter Life Sciences |
| NEB Blunt/TA Ligase Master Mix | New England Biolabs® Inc. |
| NEBNext End repair | New England Biolabs® Inc. |
| 1.5 mL Eppendorf tubes | Thermo Fisher Scientific |
| Invitrogen™ Nuclease-free water | Thermo Fisher Scientific |
| NEBNext® Quick Ligation Reaction Buffer | New England Biolabs® Inc. |
| NEBNext FFPE Repair Mix | New England Biolabs® Inc. |
| T4 DNA Ligase 2M U/mL | New England Biolabs® Inc. |
| 0.2 mL thin-walled PCR tubes | Thermo Fisher Scientific |
| Ethanol abs. | Merck |

**Table S1** **Materials & consumables**

Note: Material used for nanopore sequencing.
