# Supplementary material for: Systematic Cross-biospecimen Evaluation of DNA Extraction Kits for Long- and Short-read Multi-metagenomic Sequencing Studies
Source: Genomics Proteomics Bioinformatics. 2022 Jun 6;20(2):405–17. doi: 10.1016/j.gpb.2022.05.006 (PMC9684153; doi:10.1016/j.gpb.2022.05.006)
Supplement: Supplementary Table S2 [file mmc4.docx]

| **Sample_ID** | **β [ng/µl] after gDNA isolation** | **V [µl] after gDNA isolation** | **Β [ng/µl] barcoded DNA** |
| --- | --- | --- | --- |
| QMK Bile | 15 | 10 | 12.7 |
| QMK Saliva | 22.3 | 38 | 19.9 |
| QPS Bile | 10 | 14 | 12 |
| QPS Saliva | 278 | 20 | 91.6 |

**Table S2 DNA concentrations of human DNA samples**

Note: Measurements taken with Nanodrop 2000 before and after barcode ligation of each sample, 5 µl barcoded DNA were pooled for adapter ligation. QPS, Qiagen DNeasy PowerSoil Pro; QMK, QiAamp DNA Microbiome Kit.
