# Supplementary material for: Systematic Cross-biospecimen Evaluation of DNA Extraction Kits for Long- and Short-read Multi-metagenomic Sequencing Studies
Source: Genomics Proteomics Bioinformatics. 2022 Jun 6;20(2):405–17. doi: 10.1016/j.gpb.2022.05.006 (PMC9684153; doi:10.1016/j.gpb.2022.05.006)
Supplement: Supplementary Table S3 [file mmc5.docx]

| **β [ng/µl] pooled barcoded DNA before adapter ligation** | **β [ng/µl] pooled barcoded DNA after adapter ligation** |
| --- | --- |
| 17.0 | 15.4 |

**Table S3 DNA concentrations of DNA library**

Note: DNA concentration of pooled barcoded DNA before and after ligation, measured with Nanodrop 2000.
